# Supplementary material for: Excited State Kinetics of Benzo[a]pyrene Is Affected by Oxygen and DNA
Source: Molecules. 2023 Jul 7;28(13):5269. doi: 10.3390/molecules28135269 (PMC10343676; doi:10.3390/molecules28135269)
Supplement: Supplementary file 1 [file molecules-28-05269-s001.zip › molecules-2473551-supplementary.pdf]

Supporting information for:

Excited State Kinetics of Benzo[a]pyrene Is Affected by Oxygen and DNA

Yunxia Han[1], Xueli Wang[1*], Xiaoxiao He[1], Menghui Jia[1], Haifeng Pan[1] and Jinquan Chen[1,2*]

1. State Key Laboratory of Precision Spectroscopy, East China Normal University, Shanghai 200241, China

2. Collaborative Innovation Center of Extreme Optics, Shanxi University, Taiyuan, Shanxi 030006, China

*Corresponding author E-mail: xlwang@lps.ecnu.edu.cn; jqchen@lps.ecnu.edu.cn

1. Additional experimental results:

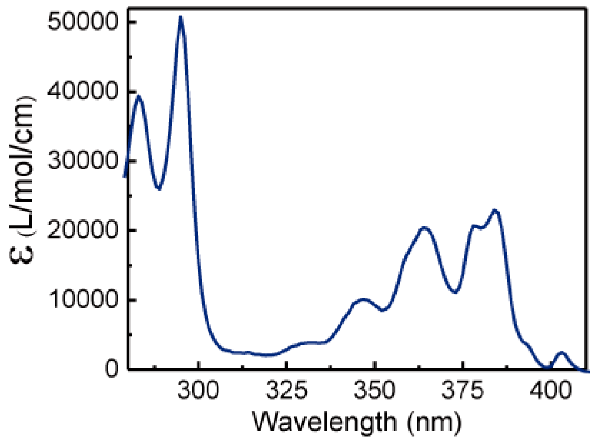

Figure S1. Steady-state absorption spectra of benzo[a]pyrene in acetonitrile.

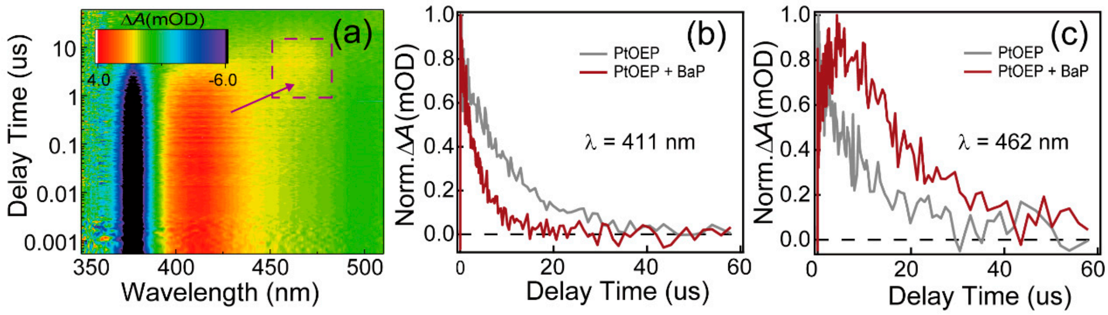

Figure S2. (a) Microsecond transient absorption spectra of mixture consisting of PtOEP and benzo[a]pyrene. (b-c) Comparison of representative Kinetic trace of PtOEP at specific wavelengths in the absence and presence of benzo[a]pyrene.

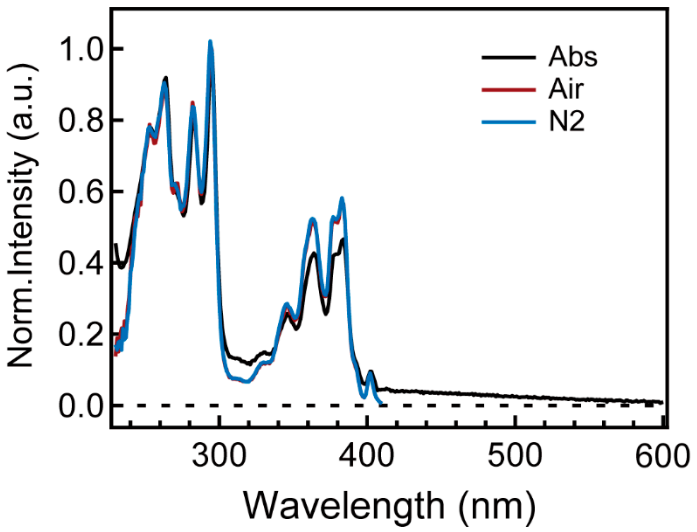

Figure S3. Comparison of absorption spectra and excitation spectra of benzo[a]pyrene in acetonitrile under air- and N_2-saturated conditions.

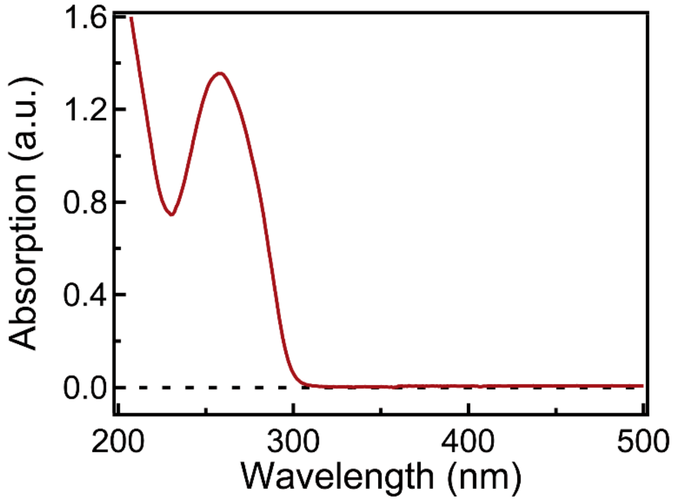

Figure S4. Steady-state absorption spectra of the mixture of benzo[a]pyrene and ct-DNA in Tris-HCL buffer.

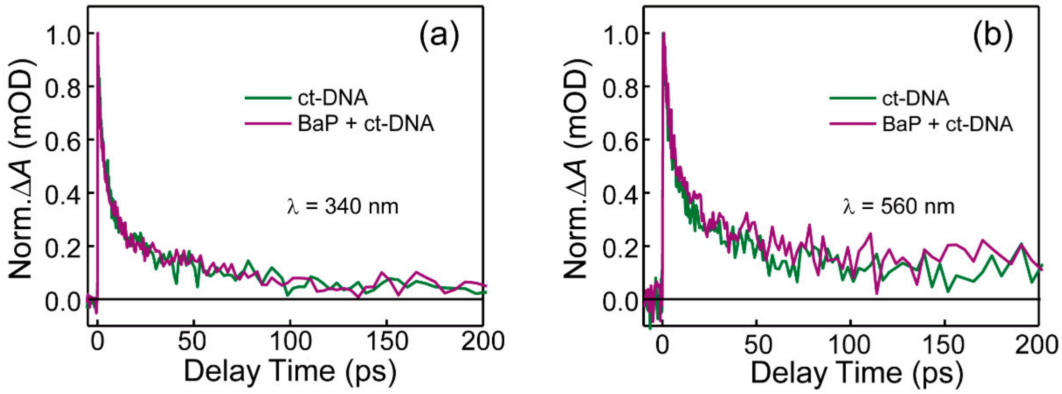

Figure S5. Comparison of representative kinetic trace of mixture consisting of ct-DNA and benzo[a]pyrene at specific probe wavelengths.

Radiative rate constant

Radiative and fluorescence lifetimes were estimated using the Strickler–Berg (SB) Equation. (S1)[1].

$k_{SB}=\frac{1}{N_A}8\pi n^2 c(2302\langle\nu_f^{-3}\rangle^{-1})\oint\frac{\varepsilon(\nu)}{\nu}d\nu$ (S1)

It can be simplified to the following equation

$k_{SB}=2.880\times 10^{-9}n^2\frac{Z_a}{Z_b}\frac{\int F(\nu)d\nu}{\int c^{-3}F(\nu)d\nu}\int\frac{\varepsilon(\nu)d\nu}{\nu}$

$=2.880\times 10^{-9}\times(1.343)^2\times 1\times\frac{4.53\times 10^{9}}{3.43\times 10^{-10}}\times 2.31\times 10^{-4}$

$=1.58\times 10^{7}s^{-1}$

Triplet quantum yield calculation

The triplet quantum yield of BaP was determined via the relative actinometry method. Experiments were run back to back with BaP and a triplet reference (Benzophenone, 100% triplet yield) standard under similar conditions at the same excitation wavelength. The BaP triplet quantum yield was calculated according to the following Equation (S2):

$$\phi_T(BaP)=\frac{\Delta A_{BaP}(\lambda_2)\phi_T(BP)\varepsilon_{BP}^*(\lambda_1)}{\Delta A_{BP}(\lambda_1)\varepsilon_{BaP}^*(\lambda_2)} \quad (S2)$$

Here, the ground state bleaching signal of BP cannot be seen in our detection window, so we use Equation (S3) to calculate the concentration of initial single excited state molecules of BP.

$$[s_{sample}^*]=\frac{(photons/pulse)(1-I/I_0)}{N_A V} \quad (S3)$$

where the number of photons per excitation pulse (380 nm) is:

$$\frac{photons}{pulse}=\frac{power}{(rep\ rate)(energy\ per\ photo)}$$

And V= Area $\times$ d

Thus

$[s^*_{sample}]=\frac{(photons/pulse)(1-I/I_0)}{N_A V}$ = 4.198 μM

The triplet extinction coefficient of BP is 7220 ± 320 $M^{-1}cm^{-1}$ (λ = 530 nm) and its triplet quantum yeild is 1 [2,3].The ground state extinction coefficient of BaP is 18371 $M^{-1}cm^{-1}$. So the triplet excited state extinction coefficient of BaP can be calculated as :

$\Delta A_{GSB(\lambda_1)}=-\varepsilon_{\lambda_1}[^3M^*]l$

$\Delta A_{T-T(\lambda_2)}=\varepsilon_{(T-T)\lambda_2}[^3M^*]l$

The average intensity of the specific GSB signal at 367 nm is around -3.18 $\times 10^{-6}$OD and the specific ESA signal at 462 nm is 3.879 $\times 10^{-6}$OD at t = 60 ns for BaP. So we obtained triplet excited state extinction coefficient of BaP is 22409 $M^{-1}cm^{-1}$.

Thus, $\phi_T(BaP)=\frac{\Delta A_{BaP}(\lambda_2)\phi_T(BP)\varepsilon^*_{BP}(\lambda_1)}{\Delta A_{BP}(\lambda_1)\varepsilon^*_{BaP}(\lambda_2)} = \frac{3.879\times10^{-6}\times7720}{22409\times5.7\times10^{-6}}$ = 23.4%

References

1. Strickler, S.J.; Berg, R.A. Relationship between Absorption Intensity and Fluorescence Lifetime of Molecules. *J. Chem. Phys.* **2004**, *37*, 814–822

2. Compton, R.H.; Grattan, K.T.V.; Morrow, T. Extinction coefficients and quantum yields for triplet—triplet absorption using laser flash photolysis. *J. Photochem.* **1980**, *14*, 61–66

3. Cuquerella, M.C.; Lhiaubet-Vallet, V.; Cadet, J.; Miranda, M.A. Benzophenone photosensitized DNA damage. *Acc. Chem. Res.* **2012**, *45*, 1558–1570
